# Causality between Autism Spectrum Disorder and Telomere Length

**DOI:** 10.1002/brb3.70362

**Published:** 2025-02-19

**Authors:** Tianyu Jin, Ruiyao Yang, Yifan Cheng, Zheng Cao, Zitian He, Shunyuan Guo

**Affiliations:** ^1^ Center for Rehabilitation Medicine, Department of Neurology Zhejiang Provincial People's Hospital, Affiliated People's Hospital, Hangzhou Medical College Hangzhou China; ^2^ Department of Rehabilitation Medicine the Second Affiliated Hospital and Yuying Children's Hospital of Wenzhou Medical University Wenzhou China; ^3^ China Rehabilitation Research Center Beijing Bo'ai Hospital Beijing China; ^4^ Department of Medicine and Health University of Sydney Sydney Australia

**Keywords:** autism spectrum disorder, causality, genetics, Mendelian randomization, telomere length

## Abstract

**Background:**

The association between telomere length (TL) and autism spectrum disorder (ASD) has received much attention. However, previous observational studies have yielded inconclusive evidence regarding this relationship. Our study aims to elucidate the causal relationship between TL and ASD using bidirectional Mendelian randomization (MR).

**Methods:**

We employed the largest genome‐wide association studies (GWAS) summary statistics for TL (sample size = 472,174) and ASD (sample size = 46,351). The primary MR analysis method was the inverse‐variance weighted (IVW) method, complemented by the MR‐Egger method, weighted median (WM) method, and MR‐PRESSO. Additionally, sensitivity analyses including Cochran's *Q* test, the intercept of MR‐Egger regression, the global test of MR‐PRESSO, and the leave‐one‐out analysis were conducted in our study.

**Results:**

The primary MR analysis indicated a significant association between ASD and shorter TL (IVW: OR = 0.98, 95% CI: 0.96–0.99, *p* = 0.03). However, no significant association was found in the reverse direction MR analysis (IVW: OR = 1.06, 95% CI: 0.94–1.23, *p* = 0.35). Raw and outlier‐corrected MR estimates from MR‐PRESSO were consistent with the IVW results. Sensitivity analyses confirmed the robustness of these findings.

**Conclusions:**

Our study indicated that individuals with ASD have shorter TL, however, shorter TL does not appear to increase the risk of ASD.

## Graphical abstract

Autism spectrum disorder is associated with shorter telomere length, but shorter telomeres do not increase the risk of ASD, based on bidirectional Mendelian randomization analysis using large GWAS datasets.

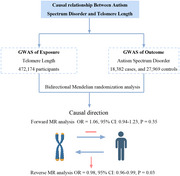

## 1 Introduction

Autism spectrum disorders (ASD) encompass a range of neurodevelopmental conditions characterized by persistent deficits in social interaction and communication, as well as restricted and repetitive patterns of behavior, interests, or activities (Lord et al. 2018). In 2020, the Centers for Disease Control and Prevention (CDC) reported that approximately one in fifty‐four children in the United States were diagnosed with ASD, a considerable increase from the 2000 estimate of one in one hundred and fifty children (Baxter et al. 2015; Hossain et al. 2020). This upward trend highlights the importance of ASD as a public health consideration, as it presents unique challenges for individuals and society. Moreover, individuals with ASD frequently have a higher probability of encountering social and economic challenges (Solmi et al. 2022). Up to now, the risk factors for ASD are complex and multifaceted, including genetic predispositions, prenatal and perinatal factors, and potentially environmental components, indicating a multifaceted etiology of the disorder (Chaste and Leboyer 2012; Vorstman et al. 2017).

Telomeres are nucleoprotein structures located at the termini of eukaryotic chromosomes and play a pivotal role in the maintenance of genomic stability (Shore 1998). These unique chromosomal components, composed of TTAGGG repeats and associated proteins, function as a molecular clock, regulating cellular lifespan and homeostasis (Tham et al. 2023). Notably, abnormal telomere length (TL), whether excessively short or long, is associated with several conditions’ states whether overly short or long. Previous studies have identified TL as an important biological marker of several neurological and psychiatric conditions (Rodríguez‐Fernández et al. 2022; Rodríguez‐Fernández, Vilor‐Tejedor, et al. 2022). Recently, there has been considerable interest in research exploring the association between telomere shortening and the risk of ASD in children(Rodríguez‐Fernández, Vilor‐Tejedor, et al. 2022; Salem and Ashaat 2023; Zhang et al. 2023). Although these studies are interesting, most of them are observational and cannot avoid their inherent limitations, such as reverse causality and confounding factors. Therefore, the causality between ASD and TL remains to be established.

The Mendelian randomization (MR) analysis is an effective strategy in epidemiological research, particularly for inferring causal relationships (Bowden and Holmes 2019). It is based on the progress and evolution of the Human Genome Project, employing genetic variants as instrumental variables (IVs) to prevent observational study limitations (Sekula et al. 2016). These IVs usually utilize single nucleotide polymorphisms (SNPs) extracted from large‐scale genome‐wide association studies (GWASs) (Wang, Cordell, and Van Steen 2019). According to Mendel's law of independent assortment, IVs can be interpreted as genetic randomized controlled trials (RCTs), likely avoiding issues of residual confounding factors and reverse causality (Davey Smith and Hemani 2014).

In this research, we employed a bidirectional two‐sample MR study to investigate the genetic causal relationships between TL and ASD using the data obtained from large‐scale GWAS.

## 2 Methods

### 2.1 Ethical Approval

This MR study used the largest published and publicly available GWAS datasets. Each participant received ethical approval and informed consent for the respective study, as detailed in the original publication and consortium.

### 2.2 Study Design

**FIGURE 1:**
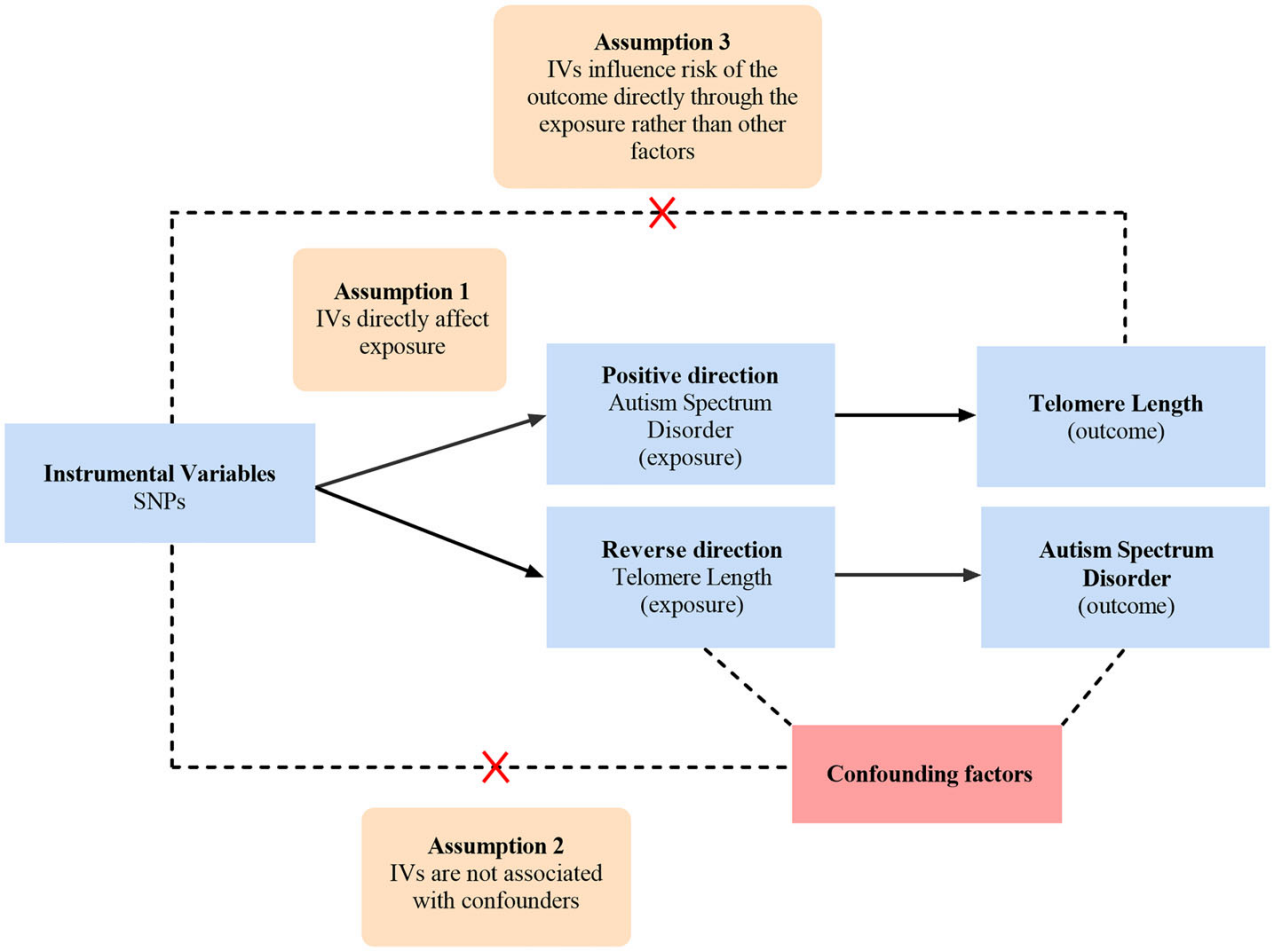
An overview of the study design.

In an attempt to assess the causal relationship between TL and ASD, we used the largest, publicly available GWAS database to conduct a bidirectional two‐sample MR analysis. ASD and TL were treated as exposures or outcomes. The MR methodology is based on the following three hypotheses (Emdin, Khera, and Kathiresan 2017): (1) the genetic instruments are associated with the exposure; (2) the genetic instruments are not linked to any confounders of the exposure‐outcome relationship; (3) the genetic instruments influence the outcome only through the exposure. Figure 1 illustrates our research design.

### 2.3 Data Sources for ASD and TL

The genetic association summary statistics for ASD were extracted from the iPSYCH‐PGC consortium. This dataset comprised 46,351 individuals of European descent, including 18,382 cases, and 27,969 controls. Participants were genotyped based on case cohort samples and a total of 9,112,386 SNPs were identified. The diagnosis of ASD was determined according to the International Classification of Diseases, Tenth Revision (ICD‐10) criteria (Grove et al. 2019). Summary data on TL were sourced from the UK Biobank, comprising a cohort of 472,174 participants with leukocyte TL measurements and 20,134,421 identified SNPs. The data were adjusted for several factors, such as age, gender, and ethnicity (Codd et al. 2021).

### 2.4 Criteria for Selecting Genetic Variants for IVs

In this MR study, we used SNPs as IVs to estimate causal effects. To ensure the robustness of these genetic IVs, we adopted stringent selection criteria. (1) SNPs reaching a genome‐wide significance threshold of P < 5×10^−8^ and demonstrating independence through linkage disequilibrium (LD, *r* ^2^< 0.001) were considered (Bowden and Holmes 2019); (2) SNPs with an F‐statistic exceeding 10 were selected to minimize bias from weak IVs. The computation of the F‐statistic is outlined in Table S1 (Burgess and Thompson 2011); (3) SNPs with a minor allele frequency (MAF) below 0.01 were excluded; (4) Ambiguous SNPs characterized by nonconcordant alleles and palindromic SNPs with indeterminate strands were either corrected or directly discarded from the chosen instrumental SNPs during the harmonization process(Bowden and Holmes 2019); (5) We employed PhenoScanner V2 to remove SNPs associated with confounding factors, ensuring the impact of SNPs on the exposure corresponded to the same allele influencing the outcome (Kamat et al. 2019).

### 2.5 Statistical Analysis

Initially, we harmonized the dataset by eliminating palindromic sequences and incompatible SNPs. Subsequently, four MR methods were used to explore the causality between TL and ASD. These methods include the inverse variance weighted (IVW) method, weighted median (WM) method, MR‐Egger method, and MR pleiotropy residual Sum and Outlier (MR‐PRESSO) method(Burgess and Thompson 2017; Li et al. 2022). The IVW method is the most commonly used in MR analysis. It estimates an estimate of the causal effect by performing a weighted average of the ratio estimates from each genetic variant, with weights being the inverse of the variance of the ratio estimate. This method assumes that all genetic variants are valid IVs, meaning they are associated with the exposure, not associated with the outcome (except through the exposure), and not associated with confounders of the exposure‐outcome relationship. Therefore, the primary method in our MR analysis is the IVW method (Wu et al. 2020). The WM method provides a consistent estimate of the causal effect even when up to 50% of the information comes from invalid IVs. It is more robust to violations of the IV assumptions than the IVW method (Loh, Noordam, and Christodoulides 2021). The MR‐Egger regression provides a test for horizontal pleiotropy and offers an estimate of the causal effect that is not biased by horizontal pleiotropy, under the assumption that the pleiotropic effects of the genetic variants are not associated with the genetic variants' associations with the exposure (Burgess and Thompson 2017). The MR‐PRESSO method detects outliers that may induce pleiotropic effects. By removing these outliers, MR‐PRESSO can provide a corrected causal effect estimate. Therefore, the MR‐PRESSO method facilitates testing for and correcting horizontal pleiotropy, where genetic variants have multiple effects (Li et al. 2022).

We conducted sensitivity analyses using various methods to ensure the reliability and stability of our results. Cochran's *Q* statistic was used to test for heterogeneity in the causal estimates derived from different genetic variants, which significant heterogeneity (*p *< 0.05) may indicate that the IV assumptions are violated for some genetic variants (Bowden and Holmes 2019). The intercept from the MR‐Egger regression served as a marker for horizontal pleiotropy, with a *p*‐value less than 0.05 suggesting its existence (Burgess and Thompson 2017). A significant intercept indicates that the genetic variants have average pleiotropic effects on the outcome, suggesting that some genetic variants might be invalid IVs. The leave‐one‐out analysis involves repeating the MR analysis multiple times, each time leaving out one genetic variant. If the MR estimate changes substantially when a specific variant is removed, this may indicate that the variant is an outlier or has a disproportionate influence on the results (Cheng, Garrick, and Fernando 2017). Additionally, a funnel plot is a scatter plot of the causal effect estimates from each genetic variant against some measure of the precision of the estimate. In the absence of directional pleiotropy, the plot should be symmetric around the overall MR estimate. Asymmetry in the funnel plot can be indicative of directional pleiotropy.

## 3 Results

### 3.1 Genetic Instruments Variables

For IVs of ASD, we selected SNPs demonstrating genome‐wide statistical significance (*p* < 5 × 10^−8^) and independence (LDr^2^< 0.001). However, this approach yielded only a limited number of eligible SNPs (*n* = 2) for ASD. To thoroughly investigate the potential causal relationship, we relaxed the statistical significance threshold to 5 × 10^−7^ (Zhao et al. 2023). Subsequently, we eliminated SNP rs2224274 through PhenoScanner V2 due to its association with age (Gao et al. 2022), and SNP rs6701243 was excluded because of its palindromic nature with intermediate allele frequencies. We finally identified 6 sets of SNPs with ASD as exposure and TL as the outcome.

For IVs of TL, we identified 154 significant and independent SNPs from the GWAS database to serve as genetic IVs. SNP rs2763979 associated with ASD was eliminated via PhenoScanner V2, as it was associated with a confounding factor (schizophrenia). Furthermore, we excluded several SNPs that were palindromic with intermediate allele frequencies and incompatible alleles. Finally, we obtained 116 sets of SNPs with TL as the exposure and ASD as the outcome.

All IVs exhibited an F‐statistic greater than 10, indicating the absence of weak genetic instruments that could potentially affect the MR estimates. Table S1 shows the exposure SNPs for ASD and TL.

### 3.2 Two‐sample MR Analysis for Causal Association of ASD and TL

**FIGURE 2:**
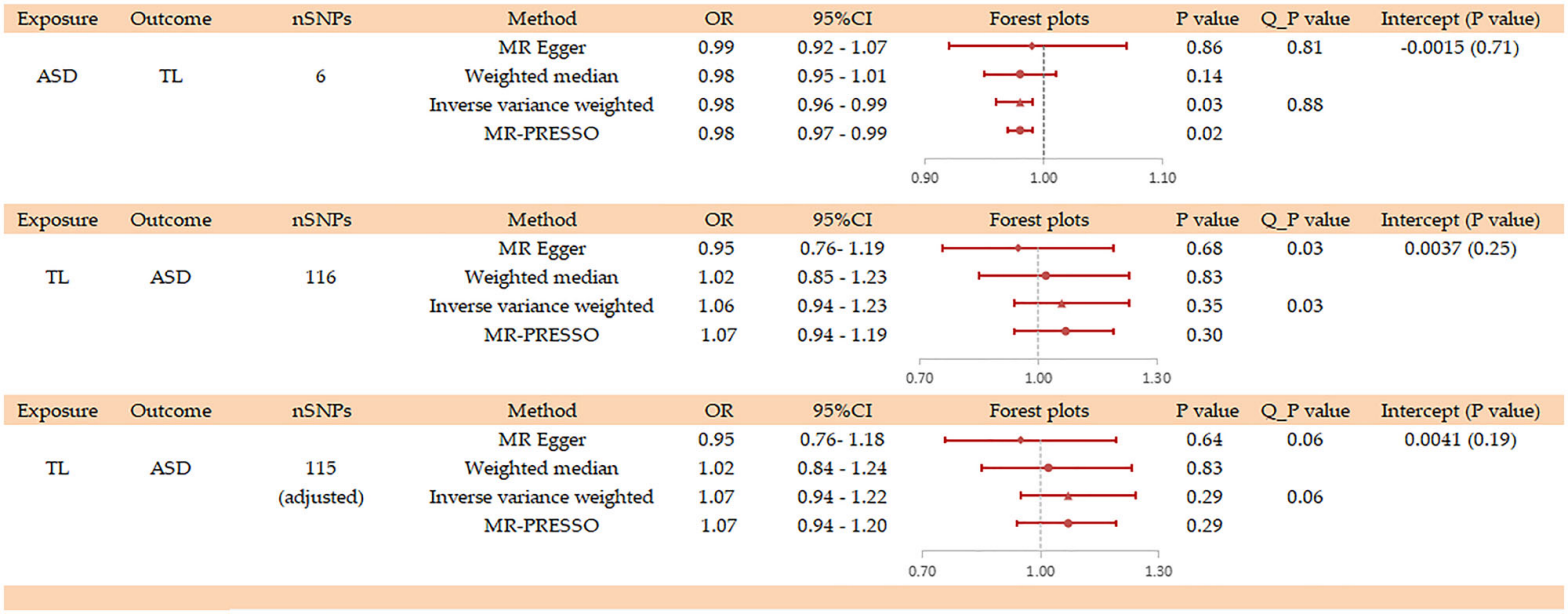
Mendelian randomization estimates of the bidirectional association between autism spectrum disorders and telomere length.

Different conclusions were yielded from the four analytical methods in our study. The primary method, the IVW method, indicated a significant genetic association between ASD and TL (OR = 0.98, 95% CI: 0.96–0.99, *p* = 0.03) (Figure 2; Figure S1A). Similarly, the MR‐PRESSO method exhibited a statistically significant association (OR = 0.98; 95% CI: 0.97–0.99, *p* = 0.02; Figure 2; Table S3). However, both the MR‐Egger method (OR = 0.99, 95% CI: 0.92–1.07, *p* = 0.86) and the WM method (OR = 0.98, 95% CI: 0.95–1.01, *p* = 0.14) did not reveal significant associations (Figure 2, Figure S1C). No significant heterogeneity (Cochran's Q_*p* = 0.88) or horizontal pleiotropy (*p* for intercept = 0.71 and global test *p* = 0.90) was detected in this MR analysis (Figure 2; Figure S1C; Table S2). Given the absence of significant heterogeneity or horizontal pleiotropy, we considered IVW results to be more reliable. Furthermore, the leave‐one‐out analysis and funnel plot confirmed the robustness of our findings (Figure S1B; Figure S1D).

### 3.3 Reverse Direction Analysis

A supplementary reverse direction MR study was conducted, using TL as the exposure and ASD as the outcome, to explore the potential of reverse causality. Our results did not demonstrate a significant causal relationship between TL and the risk of ASD when applying IVW method (OR = 1.06, 95% CI: 0.94–1.23, *p* = 0.35), MR‐Egger method (OR = 0.95, 95% CI: 0.76–1.19, *p* = 0.68), WM method (OR = 1.02, 95% CI: 0.85–1.23, *p* = 0.83), and MR‐PRESSO method (OR = 1.07, 95% CI: 0.94–1.19, *p* = 0.30; Figure 2; Figure S2A,C; Table S3). Notably, Cochran's Q statistic revealed significant heterogeneity (*p* = 0.03; Table S2). After using the MR‐PRESSO method, an outlier (rs12369950) was identified and subsequently eliminated from the analysis, resulting in nonsignificant heterogeneity (*p* = 0.06; Table S2). However, the causal relationship between TL and ASD risk remained unsupported (IVW: OR = 1.07, 95%CI: 0.94–1.22, *p* = 0.29) (Figure 2; Figure S3A). The MR‐Egger intercept showed no significant horizontal pleiotropy (*p* = 0.20 and adjusted *p* = 0.19; Figure S3C). The reliability of our results was further supported by leave‐one‐out analysis and funnel plot (Figures S2B,D and S3B,D).

## 4 Discussion

This is the first bi‐directional MR analysis examining the genetic association between ASD and TL using the largest GWAS database. Our findings suggest a significant genetic association, indicating that ASD individuals have shorter TL, however, our study did not find evidence that TL affects the risk of ASD.

Until now, research on ASD and TL has been limited. The findings from existing studies generally align with our findings. For instance, one study investigated the relationship between relative telomere length (RTL) in peripheral blood leukocytes and ASD, demonstrating that individuals with childhood ASD have significantly shorter RTL compared with controls (Salem and Ashaat 2023). Notably, shorter RTLs were found in male individuals but not in female individuals, suggesting a sexually dimorphic pattern in the molecular physiology of ASD. This may provide a molecular explanation for the higher prevalence of ASD in males (Panahi et al. 2023). Additionally, another study reported a connection between shortened telomeres and increased sensory symptoms in individuals with ASD, indicating that TL may influence not only the presence of ASD but also the severity and nature of its symptoms (Lewis et al. 2020). However, the relationship appears to be complex, as studies have shown no strong associations between RTL and other clinical features such as parental age, age of onset, illness duration, and certain ASD severity scores (Li et al. 2014). This complexity highlights the necessity for further research into understanding these relationships in more detail. Interestingly, this study showed that family training interventions could significantly affect TL in individuals with ASD. This introduces the idea that environmental factors or lifestyle modifications might influence these genetic markers, which could potentially have significant implications for ASD treatment approaches (Li et al. 2014). In our MR study, we minimized the influence of these confounding factors to obtain more accurate causal associations.

Our study's findings indicate that individuals with ASD exhibit shorter TL. Several potential mechanisms may underlie this association: Research indicates that individuals with ASD often face significant challenges related to societal acceptance, social integration, and adaptation to everyday environments (Wisner‐Carlson, Uram, and Flis 2020). These challenges manifest in higher levels of social isolation, societal misunderstandings, and stigma, all recognized sources of chronic stress that can negatively impact both psychological well‐being and biological aging processes (Efe, Aksoy, Ok, Kocak, and Gunes 2024; Stein et al. 2018). Specifically, societal misunderstandings, lack of appropriate accommodations in educational and workplace settings, and insufficient support systems exacerbate feelings of exclusion and anxiety, potentially leading to the activation of stress pathways such as the hypothalamic‐pituitary‐adrenal axis (Modabbernia, Velthorst, and Reichenberg 2017). These persistent stressors trigger physiological responses that accelerate telomere attrition over time. Chronic stress—whether social, emotional, or environmental—induces increased oxidative stress, thereby accelerating telomere shortening (Rose et al. 2012; Salim 2016). Additionally, chronic inflammation, characterized by elevated levels of inflammatory markers, promotes cellular turnover and subsequent telomere shortening (Masi et al. 2011; Zhang et al. 2023). Studies have identified elevated levels of inflammatory markers in individuals with ASD, suggesting persistent inflammatory states that can promote cellular turnover and further telomere erosion (Aulinas et al. 2015; Masi et al. 2011). The inflammatory process often necessitates cellular proliferation for repair and maintenance, inherently leading to telomere erosion (Lin et al. 2018). Beyond these factors, individuals with ASD may also experience disrupted sleep patterns and irregular circadian rhythms, which have been linked to increased oxidative stress and inflammation, further contributing to telomere shortening (Johnson and Zarrinnegar 2024). Additionally, lifestyle factors such as lower levels of physical activity and poorer dietary habits, which are sometimes observed in individuals with ASD, can exacerbate oxidative stress and inflammatory responses, thereby accelerating cellular aging (Dhaliwal et al. 2019; Yap et al. 2021).

The implications of shortened TL in individuals with ASD are profound, as reduced TL is associated with increased risk for age‐related diseases, such as cardiovascular disease, diabetes, and certain cancers, as well as diminished immune function and cognitive decline (Crespi 2011; Jin et al. 2024). This accelerated cellular aging may contribute to the reduced life expectancy and increased comorbidity rates observed in the ASD population. Addressing the multifaceted sources of chronic stress in individuals with ASD is crucial for mitigating their impact on cellular aging. Interventions aimed at improving social integration, reducing stigma, and providing adequate support and accommodations can help alleviate stress levels. Additionally, promoting healthy lifestyle behaviors, such as regular physical activity, balanced nutrition, and proper sleep hygiene, may further protect against oxidative stress and inflammation, thereby preserving TL.

The primary strength of our study is the application of MR analyses using the comprehensive GWAS database to explore bidirectional causality between ASD and TL. This methodology effectively avoids the confounding factors and reverse causality issues present in previous observational studies. Additionally, we carefully screened the selected SNPs using Plink clump and Phenoscanner V2. All F‐statistics were greater than 10, thereby eliminating the bias associated with weak IVs bias. However, our study has some limitations. First, we chose a loose SNP inclusion threshold of 5 × 10^−7^ due to few genome‐wide significant SNPs for ASD, and there remains a necessity for more extensive GWAS databases to facilitate future study. Second, our analysis was restricted to GWAS data at the abstract level, which prevented more detailed subgroup analyses based on demographic data, clinical symptoms, and individual ASD subtypes. Third, the findings from MR studies may not be generalizable, as the sample was obtained from the European population, further exploration is necessary for other demographic groups. Fourth, inherent limitations of MR analysis, such as RNA editing and inactive transposons, could not be addressed in our study.

## 5 Conclusion

In conclusion, our study offers genetic evidence linking ASD to TL, suggesting that individuals with ASD may have shorter telomeres. These findings identify potential areas for future research. Additional research is necessary to elucidate the role of shorter TL in ASD, particularly regarding its implications for early diagnosis, symptom severity, and treatment interventions.

## Author Contributions

**Tianyu Jin**: Conceptualization, data curation, formal analysis, methodology, software, writing–review and editing, and writing–original draft. **Ruiyao Yang**: Writing–original draft, and writing–review and editing. **Yifan Cheng**: Writing–review and editing, validation, and visualization. **Zheng Cao**: Writing–review and editing, visualization, and validation. **Zitian He**: Writing–review and editing, visualization, and validation. **Shunyuan Guo**: Writing–review and editing, validation, supervision, and funding acquisition.

## Conflicts of Interest

The authors declare no conflicts of interest.

## Ethics Approval and Consent to Participate

The database for ASD received approval from the Regional Scientific Ethics Committee in Denmark and the Danish Data Protection Agency. Additionally, the database for TL obtained approval from the North West Multi‐centre Research Ethics Committee.

## Consent for Publication

No conflicts of interest exist in the submission of this manuscript. All authors approved the manuscript for publication.

### Peer Review

The peer review history for this article is available at https://publons.com/publon/10.1002/brb3.70362

## Supporting information



Supporting Information

Supporting Information

Supporting Information

Supporting Information

Supporting Information

## Data Availability

The datasets generated during the current study are available in the iPSYCH‐PGC repository for ASD and the UK Biobank repository for TL. These datasets are publicly available and can be found at the following URLs: https://www.nature.com/articles/s41588‐019‐0344‐8 and https://www.nealelab.is/uk‐biobank. The data that supports the findings of this study are available in the supplementary material of this article.
