# Supplementary material for: Causality between Autism Spectrum Disorder and Telomere Length
Source: Brain Behav. 2025 Feb 19;15(2):e70362. doi: 10.1002/brb3.70362 (PMC11839737; doi:10.1002/brb3.70362)
Supplement: Supplementary file 1 — Supporting Information [file BRB3-15-e70362-s004.docx]

Supplementary Materials

**Supplementary Table 1.** SNPs from GWAS on autism spectrum disorder and telomere length.

**Supplementary Table 2.** Heterogeneity of MR analysis for autism spectrum disorder and telomere length.

**Supplementary Table 3.** Associations of genetic prediction of autism spectrum disorder and telomere length in the MR-PRESSO analysis.

**Supplementary Figure 1. A.** MR estimates of the association between ASD and TL. We found a significant genetic association between ASD and TL (OR = 0.98, 95% CI: 0.96-0.99, P = 0.03). **B.** Leave-one-out plots of ASD and TL. The leave-one-out plot visualizes how the causal estimates (point with horizontal line) for the effect of ASD on TL are influenced by the exclusion of individual SNPs. We did not find that a particular SNP had a significant effect on the results. **C.** Scatter plot of the association between ASD and TL. Three lines reveal the estimated effect sizes by MR methods (inverse‐variance weighted, MR-Egger and weighted median). **D.** Funnel plot on ASD and TL. The funnel plots are symmetric, which shows that the absence of polymorphism.

**Supplementary Figure 2. A.** MR estimates of the association between TL and ASD. We did not find a significant genetic association between ASD and TL (OR = 1.06, 95% CI: 0.94-1.23, P = 0.35). B. Leave-one-out plots of TL on ASD. We did not find that a particular SNP had a significant effect on the results. **C.** Scatter plot of the association between ASD and TL. **D.** Funnel plot of TL on ASD. The funnel plots are symmetric, which shows that the absence of polymorphism.

**Supplementary Figure 3. A.** MR estimates of the association between TL and ASD after MR-PRESSO adjustment. We still did not find a significant genetic association between ASD and TL (adjusted, OR = 1.07, 95% CI: 0.94-1.22, P = 0.29). B. Leave-one-out plots of TL on ASD (adjusted). We did not find that a particular SNP had a significant effect on the results. **C.** Scatter plot of the association between ASD and TL (adjusted). **D.** Funnel plot of TL on ASD. The funnel plots are symmetric, which shows that the absence of polymorphism (adjusted).

**Supplementary Table 1**. SNPs from GWAS on autism spectrum disorder and telomere length.

|  | **SNP** | **Samplesize** | **EA** | **OA** | **β** | **SE** | **EAF** | **P** value | **R**2* | **F - statistic**** |
| --- | --- | --- | --- | --- | --- | --- | --- | --- | --- | --- |
| **ASD** |  |  |  |  |  |  |  |  |  |  |
|  | rs2391769 | 46351 | G | A | 0.0769 | 0.0145 | 0.3300 | 1.14E-07 | 0.00262 | 121.53 |
|  | rs6701243 | 46351 | C | A | -0.0735 | 0.0144 | 0.6412 | 3.07E-07 | 0.00249 | 115.50 |
|  | rs1452075 | 46351 | T | C | 0.0807 | 0.0155 | 0.2843 | 2.07E-07 | 0.00265 | 123.17 |
|  | rs111931861 | 46351 | G | A | 0.2169 | 0.0409 | 0.9433 | 1.12E-07 | 0.00503 | 234.43 |
|  | rs45595836 | 46351 | T | C | 0.1390 | 0.0272 | 0.9066 | 3.13E-07 | 0.00327 | 152.15 |
|  | rs112635299 | 46351 | T | G | 0.2210 | 0.0432 | 0.9831 | 3.04E-07 | 0.00162 | 75.34 |
|  | rs910805 | 46351 | A | G | -0.0957 | 0.0160 | 0.7644 | 2.04E-09 | 0.00330 | 153.39 |
| **TL** |  |  |  |  |  |  |  |  |  |  |
|  | rs3767952 | 472174 | A | G | 0.0134 | 0.0024 | 0.2267 | 1.80E-08 | 0.00006 | 29.94 |
|  | rs4498805 | 472174 | T | G | 0.0151 | 0.0020 | 0.5466 | 5.70E-14 | 0.00011 | 53.09 |
|  | rs932002 | 472174 | T | C | -0.0402 | 0.0028 | 0.1508 | 7.30E-47 | 0.00041 | 195.61 |
|  | rs66731853 | 472174 | A | G | -0.0178 | 0.0022 | 0.3173 | 1.50E-16 | 0.00014 | 64.67 |
|  | rs11584821 | 472174 | T | C | -0.0307 | 0.0026 | 0.1762 | 3.00E-31 | 0.00027 | 128.83 |
|  | rs6659669 | 472174 | T | C | -0.0117 | 0.0021 | 0.6051 | 1.10E-08 | 0.00007 | 30.94 |
|  | rs2977608 | 472174 | C | A | 0.0129 | 0.0023 | 0.7439 | 3.00E-08 | 0.00006 | 30.16 |
|  | rs6669563 | 472174 | A | G | 0.0182 | 0.0020 | 0.4378 | 2.10E-19 | 0.00016 | 77.31 |
|  | rs145114957 | 472174 | G | C | 0.0273 | 0.0050 | 0.0426 | 4.60E-08 | 0.00006 | 28.65 |
|  | rs41269079 | 472174 | A | T | 0.0154 | 0.0025 | 0.1890 | 1.70E-09 | 0.00007 | 34.16 |
|  | rs139795227 | 472174 | C | A | 0.0599 | 0.0087 | 0.0140 | 6.70E-12 | 0.00010 | 46.91 |
|  | rs11579626 | 472174 | C | A | 0.0265 | 0.0036 | 0.0849 | 1.30E-13 | 0.00011 | 51.56 |
|  | rs6587577 | 472174 | G | A | -0.0182 | 0.0026 | 0.8263 | 4.80E-12 | 0.00010 | 44.96 |
|  | rs6751209 | 472174 | C | T | -0.0140 | 0.0025 | 0.2042 | 1.60E-08 | 0.00006 | 30.28 |
|  | rs188918174 | 472174 | T | C | 0.0403 | 0.0054 | 0.0361 | 1.20E-13 | 0.00011 | 53.40 |
|  | rs202034370 | 472174 | T | TA | 0.1028 | 0.0065 | 0.9754 | 2.60E-56 | 0.00051 | 239.41 |
|  | rs56178008 | 472174 | A | T | 0.0144 | 0.0020 | 0.4375 | 9.70E-13 | 0.00010 | 48.02 |
|  | rs376641875 | 472174 | C | CATAA | -0.0266 | 0.0042 | 0.9289 | 3.30E-10 | 0.00009 | 44.10 |
|  | rs17803849 | 472174 | T | C | 0.0273 | 0.0020 | 0.4052 | 4.20E-41 | 0.00036 | 169.94 |
|  | rs2555104 | 472174 | C | A | -0.0140 | 0.0020 | 0.4343 | 6.60E-12 | 0.00010 | 45.29 |
|  | rs77732866 | 472174 | A | G | 0.0178 | 0.0029 | 0.1376 | 9.20E-10 | 0.00008 | 35.48 |
|  | rs2230590 | 472174 | C | T | -0.0158 | 0.0020 | 0.5109 | 3.60E-15 | 0.00012 | 58.93 |
|  | rs78491606 | 472174 | C | A | -0.0756 | 0.0074 | 0.0184 | 1.90E-24 | 0.00021 | 97.75 |
|  | rs9878436 | 472174 | T | C | -0.0143 | 0.0020 | 0.4344 | 1.20E-12 | 0.00010 | 47.72 |
|  | rs35446936 | 472174 | A | G | -0.0940 | 0.0023 | 0.2437 | 1.00E-200 | 0.00326 | 1542.92 |
|  | rs112394943 | 472174 | C | T | -0.0199 | 0.0028 | 0.1627 | 1.60E-12 | 0.00011 | 50.94 |
|  | rs13062095 | 472174 | C | T | 0.0139 | 0.0021 | 0.3278 | 9.70E-11 | 0.00008 | 39.95 |
|  | rs4616688 | 472174 | T | G | -0.0173 | 0.0020 | 0.5254 | 4.50E-18 | 0.00015 | 70.87 |
|  | rs6790988 | 472174 | G | A | 0.0146 | 0.0023 | 0.7419 | 1.80E-10 | 0.00008 | 38.40 |
|  | rs869785 | 472174 | C | T | -0.0147 | 0.0021 | 0.6725 | 4.40E-12 | 0.00010 | 45.14 |
|  | rs6776756 | 472174 | A | G | -0.0174 | 0.0020 | 0.5976 | 1.10E-17 | 0.00015 | 69.11 |
|  | rs11426156 | 472174 | T | TA | -0.0116 | 0.0021 | 0.3995 | 2.20E-08 | 0.00006 | 30.42 |
|  | rs35500378 | 472174 | CACTT | C | 0.0145 | 0.0021 | 0.6105 | 2.00E-12 | 0.00010 | 47.00 |
|  | rs10805346 | 472174 | C | T | 0.0117 | 0.0020 | 0.4393 | 7.00E-09 | 0.00007 | 31.88 |
|  | rs6536702 | 472174 | A | G | 0.0534 | 0.0024 | 0.7746 | 9.40E-111 | 0.00100 | 470.82 |
|  | rs871134 | 472174 | T | C | -0.0183 | 0.0020 | 0.5690 | 1.70E-19 | 0.00016 | 77.56 |
|  | rs4695407 | 472174 | G | A | 0.0142 | 0.0020 | 0.5078 | 1.50E-12 | 0.00010 | 47.27 |
|  | rs2282764 | 472174 | G | A | -0.0224 | 0.0029 | 0.1424 | 9.30E-15 | 0.00012 | 57.99 |
|  | rs7705526 | 472174 | A | C | 0.0776 | 0.0022 | 0.3266 | 1.00E-200 | 0.00265 | 1254.02 |
|  | rs73730598 | 472174 | A | G | 0.0274 | 0.0044 | 0.0548 | 4.70E-10 | 0.00008 | 36.62 |
|  | rs61748181 | 472174 | T | C | -0.0592 | 0.0060 | 0.0289 | 2.80E-23 | 0.00020 | 92.93 |
|  | rs6881568 | 472174 | A | C | 0.0169 | 0.0021 | 0.3626 | 3.70E-16 | 0.00013 | 62.53 |
|  | rs55747751 | 472174 | A | G | -0.0212 | 0.0038 | 0.0774 | 1.70E-08 | 0.00006 | 30.19 |
|  | rs185174247 | 472174 | A | G | 0.0373 | 0.0044 | 0.0561 | 1.10E-17 | 0.00015 | 69.50 |
|  | rs141214782 | 472174 | TTATC | T | -0.0247 | 0.0034 | 0.1012 | 2.00E-13 | 0.00011 | 52.28 |
|  | rs28363070 | 472174 | A | G | 0.0756 | 0.0096 | 0.0134 | 3.50E-15 | 0.00015 | 71.22 |
|  | rs1611236 | 472174 | A | G | -0.0160 | 0.0021 | 0.3269 | 6.10E-14 | 0.00011 | 53.29 |
|  | rs142730696 | 472174 | TTTTTC | T | 0.0217 | 0.0030 | 0.8641 | 6.30E-13 | 0.00011 | 52.20 |
|  | rs7772289 | 472174 | T | G | 0.0175 | 0.0020 | 0.5031 | 1.70E-18 | 0.00015 | 72.72 |
|  | rs80324517 | 472174 | A | G | 0.0397 | 0.0047 | 0.0483 | 1.80E-17 | 0.00014 | 68.20 |
|  | rs201558190 | 472174 | C | T | -0.0182 | 0.0022 | 0.3633 | 6.40E-17 | 0.00015 | 72.16 |
|  | rs9398196 | 472174 | G | A | -0.0144 | 0.0020 | 0.5201 | 9.50E-13 | 0.00010 | 48.60 |
|  | rs61405042 | 472174 | T | C | -0.0502 | 0.0060 | 0.0293 | 8.50E-17 | 0.00014 | 67.63 |
|  | rs2538745 | 472174 | C | T | -0.0129 | 0.0021 | 0.6028 | 3.10E-10 | 0.00008 | 37.87 |
|  | rs117407747 | 472174 | T | C | 0.0451 | 0.0061 | 0.0276 | 1.80E-13 | 0.00011 | 51.40 |
|  | rs1985369 | 472174 | G | A | -0.0312 | 0.0030 | 0.8682 | 3.60E-25 | 0.00022 | 105.16 |
|  | rs13230646 | 472174 | C | T | -0.0173 | 0.0023 | 0.2489 | 8.90E-14 | 0.00011 | 53.02 |
|  | rs11769630 | 472174 | A | T | -0.0257 | 0.0039 | 0.0722 | 4.30E-11 | 0.00009 | 41.74 |
|  | rs2056726 | 472174 | A | G | -0.0228 | 0.0024 | 0.2144 | 7.90E-21 | 0.00018 | 82.75 |
|  | rs7790856 | 472174 | T | C | -0.0437 | 0.0022 | 0.2891 | 1.80E-87 | 0.00079 | 371.30 |
|  | rs4731541 | 472174 | G | C | -0.0206 | 0.0021 | 0.6249 | 1.40E-23 | 0.00020 | 94.06 |
|  | rs117630647 | 472174 | A | G | 0.0596 | 0.0072 | 0.0213 | 1.40E-16 | 0.00015 | 70.00 |
|  | rs2306646 | 472174 | C | G | -0.0209 | 0.0020 | 0.5595 | 3.30E-25 | 0.00022 | 102.09 |
|  | rs762679 | 472174 | A | T | 0.0310 | 0.0029 | 0.8565 | 1.40E-27 | 0.00024 | 111.64 |
|  | rs11991877 | 472174 | A | T | -0.0301 | 0.0032 | 0.8893 | 3.20E-21 | 0.00018 | 84.45 |
|  | rs10112752 | 472174 | A | G | -0.0288 | 0.0020 | 0.4304 | 9.50E-46 | 0.00041 | 191.46 |
|  | rs1023767 | 472174 | A | G | -0.0184 | 0.0023 | 0.2376 | 5.00E-15 | 0.00012 | 57.75 |
|  | rs34896435 | 472174 | G | C | 0.0155 | 0.0021 | 0.4692 | 6.40E-14 | 0.00012 | 56.20 |
|  | rs11557154 | 472174 | T | C | -0.0344 | 0.0030 | 0.1300 | 1.10E-30 | 0.00027 | 126.24 |
|  | rs4743037 | 472174 | T | C | 0.0148 | 0.0024 | 0.2309 | 5.10E-10 | 0.00008 | 36.72 |
|  | rs117034449 | 472174 | A | G | 0.0374 | 0.0067 | 0.0233 | 2.10E-08 | 0.00006 | 30.13 |
|  | rs7099229 | 472174 | A | G | -0.0153 | 0.0022 | 0.2733 | 8.40E-12 | 0.00009 | 44.07 |
|  | rs12412214 | 472174 | A | G | -0.0245 | 0.0022 | 0.2798 | 3.40E-28 | 0.00024 | 114.41 |
|  | rs6584579 | 472174 | G | A | 0.0115 | 0.0020 | 0.3989 | 2.00E-08 | 0.00006 | 29.91 |
|  | rs9419958 | 472174 | C | T | -0.0810 | 0.0029 | 0.8614 | 2.60E-167 | 0.00157 | 741.11 |
|  | rs77231040 | 472174 | C | G | 0.0989 | 0.0135 | 0.0057 | 2.00E-13 | 0.00011 | 52.77 |
|  | rs10905255 | 472174 | T | G | -0.0182 | 0.0020 | 0.5792 | 2.60E-19 | 0.00016 | 76.67 |
|  | rs939916 | 472174 | A | G | 0.0242 | 0.0022 | 0.6700 | 6.60E-29 | 0.00026 | 122.11 |
|  | rs10840270 | 472174 | G | C | 0.0144 | 0.0021 | 0.6557 | 1.30E-11 | 0.00009 | 44.11 |
|  | rs2293579 | 472174 | A | G | -0.0129 | 0.0021 | 0.3863 | 3.30E-10 | 0.00008 | 37.34 |
|  | rs10768683 | 472174 | G | C | 0.0470 | 0.0028 | 0.8410 | 1.50E-64 | 0.00059 | 278.97 |
|  | rs11212631 | 472174 | C | T | -0.0193 | 0.0026 | 0.1992 | 4.70E-14 | 0.00012 | 56.39 |
|  | rs6590343 | 472174 | G | A | 0.0122 | 0.0020 | 0.5164 | 1.50E-09 | 0.00007 | 34.95 |
|  | rs611646 | 472174 | A | T | -0.0368 | 0.0020 | 0.4087 | 3.50E-73 | 0.00066 | 309.78 |
|  | rs12369950 | 472174 | C | T | -0.0178 | 0.0029 | 0.1407 | 8.00E-10 | 0.00008 | 36.30 |
|  | rs79977579 | 472174 | A | C | 0.0282 | 0.0034 | 0.0956 | 2.30E-16 | 0.00014 | 64.69 |
|  | rs10773176 | 472174 | G | A | -0.0172 | 0.0023 | 0.7412 | 5.20E-14 | 0.00011 | 53.60 |
|  | rs10845387 | 472174 | A | G | -0.0141 | 0.0021 | 0.3527 | 1.50E-11 | 0.00009 | 42.99 |
|  | rs17445108 | 472174 | A | G | -0.0169 | 0.0030 | 0.1270 | 2.00E-08 | 0.00006 | 29.87 |
|  | rs10774624 | 472174 | A | G | 0.0150 | 0.0021 | 0.5328 | 2.90E-13 | 0.00011 | 52.86 |
|  | rs1907702 | 472174 | A | G | 0.0150 | 0.0024 | 0.7668 | 5.90E-10 | 0.00008 | 38.13 |
|  | rs76666449 | 472174 | C | T | 0.0295 | 0.0033 | 0.1006 | 8.20E-19 | 0.00016 | 74.45 |
|  | rs28577594 | 472174 | C | G | 0.0188 | 0.0022 | 0.7098 | 5.40E-17 | 0.00015 | 68.51 |
|  | rs1332941 | 472174 | G | A | 0.0257 | 0.0027 | 0.8205 | 5.90E-21 | 0.00019 | 91.57 |
|  | rs670180 | 472174 | A | T | -0.0116 | 0.0020 | 0.5691 | 1.20E-08 | 0.00007 | 31.06 |
|  | rs9600019 | 472174 | T | C | 0.0127 | 0.0021 | 0.3356 | 2.40E-09 | 0.00007 | 34.03 |
|  | rs73581419 | 472174 | T | C | 0.0230 | 0.0032 | 0.1066 | 1.30E-12 | 0.00010 | 47.52 |
|  | rs113525195 | 472174 | A | C | -0.0124 | 0.0022 | 0.2903 | 3.10E-08 | 0.00006 | 29.95 |
|  | rs45604339 | 472174 | T | C | -0.0204 | 0.0021 | 0.3424 | 4.30E-22 | 0.00019 | 88.79 |
|  | rs137901416 | 472174 | A | G | 0.0457 | 0.0033 | 0.1003 | 4.70E-43 | 0.00038 | 178.22 |
|  | rs1957937 | 472174 | T | A | 0.0209 | 0.0027 | 0.1602 | 1.90E-14 | 0.00012 | 55.69 |
|  | rs3093888 | 472174 | A | G | -0.0290 | 0.0045 | 0.0513 | 1.50E-10 | 0.00008 | 38.59 |
|  | rs34550383 | 472174 | C | CT | -0.0192 | 0.0020 | 0.5474 | 8.60E-22 | 0.00018 | 86.48 |
|  | rs17677991 | 472174 | G | C | 0.0223 | 0.0021 | 0.3421 | 4.40E-26 | 0.00022 | 105.40 |
|  | rs5742915 | 472174 | C | T | 0.0193 | 0.0020 | 0.4458 | 1.60E-21 | 0.00018 | 87.26 |
|  | rs7164950 | 472174 | G | A | 0.0129 | 0.0020 | 0.4060 | 2.30E-10 | 0.00008 | 38.11 |
|  | rs11412296 | 472174 | T | TA | 0.0332 | 0.0023 | 0.7594 | 1.40E-45 | 0.00040 | 190.57 |
|  | rs80116508 | 472174 | A | G | -0.0353 | 0.0042 | 0.0624 | 2.00E-17 | 0.00015 | 68.68 |
|  | rs76219171 | 472174 | A | G | 0.0360 | 0.0043 | 0.0584 | 7.80E-17 | 0.00014 | 67.28 |
|  | rs2967355 | 472174 | C | A | -0.0462 | 0.0024 | 0.7743 | 4.00E-83 | 0.00074 | 351.92 |
|  | rs12925933 | 472174 | C | A | -0.0147 | 0.0021 | 0.6622 | 7.00E-12 | 0.00010 | 45.42 |
|  | rs111950327 | 472174 | C | G | 0.0238 | 0.0041 | 0.0636 | 5.90E-09 | 0.00007 | 31.94 |
|  | rs450962 | 472174 | G | A | 0.0143 | 0.0025 | 0.2838 | 5.90E-09 | 0.00008 | 39.16 |
|  | rs3785074 | 472174 | G | A | 0.0239 | 0.0022 | 0.2897 | 2.60E-27 | 0.00023 | 110.67 |
|  | rs76065543 | 472174 | T | C | 0.0343 | 0.0029 | 0.1375 | 4.20E-32 | 0.00028 | 131.70 |
|  | rs12932179 | 472174 | G | A | -0.0136 | 0.0020 | 0.5614 | 1.80E-11 | 0.00009 | 43.17 |
|  | rs182059586 | 472174 | C | T | -0.0571 | 0.0068 | 0.0251 | 4.90E-17 | 0.00016 | 75.43 |
|  | rs11117354 | 472174 | C | T | 0.0233 | 0.0022 | 0.6965 | 3.40E-26 | 0.00023 | 107.94 |
|  | rs9940099 | 472174 | T | G | -0.0336 | 0.0041 | 0.0627 | 3.20E-16 | 0.00013 | 62.71 |
|  | rs56061761 | 472174 | A | G | -0.0204 | 0.0022 | 0.3326 | 6.90E-20 | 0.00018 | 86.88 |
|  | rs4724 | 472174 | A | G | -0.0547 | 0.0031 | 0.1166 | 9.80E-69 | 0.00062 | 291.70 |
|  | rs12451892 | 472174 | C | T | -0.0116 | 0.0021 | 0.3805 | 2.20E-08 | 0.00006 | 30.03 |
|  | rs7209057 | 472174 | A | G | 0.0118 | 0.0020 | 0.5610 | 5.70E-09 | 0.00007 | 32.49 |
|  | rs59409453 | 472174 | G | A | 0.0202 | 0.0023 | 0.7306 | 1.60E-18 | 0.00016 | 75.95 |
|  | rs111527438 | 472174 | C | T | 0.0125 | 0.0021 | 0.3513 | 3.10E-09 | 0.00007 | 33.63 |
|  | rs7221585 | 472174 | T | C | 0.0143 | 0.0025 | 0.2240 | 6.70E-09 | 0.00007 | 33.70 |
|  | rs75664430 | 472174 | G | C | -0.0235 | 0.0023 | 0.2480 | 3.60E-24 | 0.00021 | 97.44 |
|  | rs56799554 | 472174 | G | A | -0.0260 | 0.0027 | 0.1702 | 3.00E-22 | 0.00019 | 90.03 |
|  | rs144204502 | 472174 | T | C | -0.1006 | 0.0091 | 0.0126 | 3.40E-28 | 0.00025 | 118.52 |
|  | rs150150565 | 472174 | T | C | 0.0638 | 0.0074 | 0.0215 | 6.80E-18 | 0.00017 | 80.62 |
|  | rs116863223 | 472174 | A | G | -0.0818 | 0.0094 | 0.0118 | 2.60E-18 | 0.00016 | 73.44 |
|  | rs16978028 | 472174 | T | A | -0.0299 | 0.0029 | 0.1437 | 8.20E-26 | 0.00022 | 104.24 |
|  | rs2276182 | 472174 | G | C | 0.0234 | 0.0020 | 0.4032 | 2.80E-30 | 0.00026 | 123.96 |
|  | rs9955360 | 472174 | A | C | -0.0190 | 0.0030 | 0.8693 | 2.20E-10 | 0.00008 | 38.87 |
|  | rs3891167 | 472174 | G | A | -0.0426 | 0.0024 | 0.2534 | 1.20E-70 | 0.00069 | 324.00 |
|  | rs139669835 | 472174 | T | C | -0.0613 | 0.0105 | 0.0094 | 6.10E-09 | 0.00007 | 32.88 |
|  | rs11085072 | 472174 | T | C | -0.0132 | 0.0024 | 0.2369 | 2.60E-08 | 0.00006 | 29.66 |
|  | rs4530278 | 472174 | T | G | 0.0139 | 0.0021 | 0.5982 | 1.50E-11 | 0.00009 | 43.73 |
|  | rs8105767 | 472174 | G | A | 0.0328 | 0.0022 | 0.2947 | 2.50E-50 | 0.00045 | 211.75 |
|  | rs429358 | 472174 | C | T | 0.0173 | 0.0028 | 0.1540 | 3.80E-10 | 0.00008 | 37.03 |
|  | rs8102497 | 472174 | A | G | -0.0150 | 0.0020 | 0.4318 | 1.40E-13 | 0.00011 | 51.90 |
|  | rs11699829 | 472174 | A | G | 0.0642 | 0.0060 | 0.0341 | 1.50E-26 | 0.00027 | 128.38 |
|  | rs142426306 | 472174 | T | C | -0.0505 | 0.0054 | 0.0395 | 8.70E-21 | 0.00019 | 91.45 |
|  | rs1291143 | 472174 | C | A | 0.0493 | 0.0028 | 0.8490 | 1.80E-69 | 0.00062 | 294.56 |
|  | rs6054257 | 472174 | A | G | -0.0142 | 0.0025 | 0.7935 | 1.10E-08 | 0.00007 | 31.06 |
|  | rs35640778 | 472174 | A | G | -0.2090 | 0.0070 | 0.0208 | 9.59E-195 | 0.00178 | 840.03 |
|  | rs143190905 | 472174 | T | G | -0.0724 | 0.0037 | 0.0804 | 1.60E-85 | 0.00078 | 366.28 |
|  | rs41304832 | 472174 | A | G | 0.0612 | 0.0093 | 0.0124 | 5.00E-11 | 0.00009 | 43.20 |
|  | rs117512405 | 472174 | A | G | -0.0790 | 0.0082 | 0.0170 | 9.50E-22 | 0.00021 | 98.79 |
|  | rs28502153 | 472174 | A | C | -0.0216 | 0.0021 | 0.3780 | 1.20E-25 | 0.00022 | 103.53 |
|  | rs6007020 | 472174 | C | T | 0.0145 | 0.0021 | 0.3678 | 4.80E-12 | 0.00010 | 46.11 |
|  | rs131797 | 472174 | T | TAAAAA | 0.0244 | 0.0024 | 0.2356 | 6.80E-25 | 0.00021 | 101.08 |
|  | rs1003322 | 472174 | A | C | 0.0142 | 0.0025 | 0.2137 | 1.00E-08 | 0.00007 | 31.88 |

SNP, single nucleotide polymorphisms; EA, effect allele; OA, other allele; SE, standard error; EAF, effect allele frequency; ASD, autism spectrum disorder; TL, telomere length.

*R^2^ =2×EAF×(1-EAF)×(β)^2^

**F - statistic=R^2^×(N-2) / (1-R^2^)

NOTE: R^2^ (The variance), the proportion of exposed variability explained by individual genetic instrument; Ν, the sample size of the exposure of GWAS.

**Supplementary Table 2.** Heterogeneity of MR analysis for autism spectrum disorder and telomere length.

| **Exposure** | **Outcome** | **Method** | **Q** | **Q_df** | **Q_P value** |
| --- | --- | --- | --- | --- | --- |
| ASD | TL | MR-Egger | 1.62 | 4 | 0.81 |
| ASD | TL | Inverse variance weighted | 1.78 | 5 | 0.88 |
| TL | ASD | MR-Egger | 143.67 | 114 | 0.03 |
| TL | ASD | Inverse variance weighted | 145.35 | 115 | 0.03 |
| TL (adjusted) | ASD (adjusted) | MR-Egger | 137.49 | 113 | 0.06 |
| TL (adjusted) | ASD (adjusted) | Inverse variance weighted | 139.60 | 114 | 0.06 |

ASD, Autism Spectrum Disorder; TL, telomere length.

**Supplementary Table 3.** Associations of genetic prediction of autism spectrum disorder and telomere length in the MR-PRESSO analysis.

| **Exposure** | **Outcome** | **MR-PRESSO** | | | | | | |
| --- | --- | --- | --- | --- | --- | --- | --- | --- |
|  |  | **SNPs** | **Outliers** | **Causal Estimate** | **SD** | **T-stat** | **P value** | **Global test P value** |
| ASD | TL | 6 | 0 | -0.0236 | 0.0068 | -3.47 | 0.018 | 0.8950 |
| TL | ASD | 116 | 1 | 0.0673 | 0.0649 | 1.04 | 0.302 | 0.0312 |
| TL | ASD | 115 (adjusted) | 0 | 0.0692 | 0.0644 | 1.07 | 0.285 | 0.0545 |

MR-PRESSO, MR-Pleiotropy Residual Sum and Outlier; SNP, single nucleotide polymorphisms; ASD, Autism Spectrum Disorder; TL, telomere length.


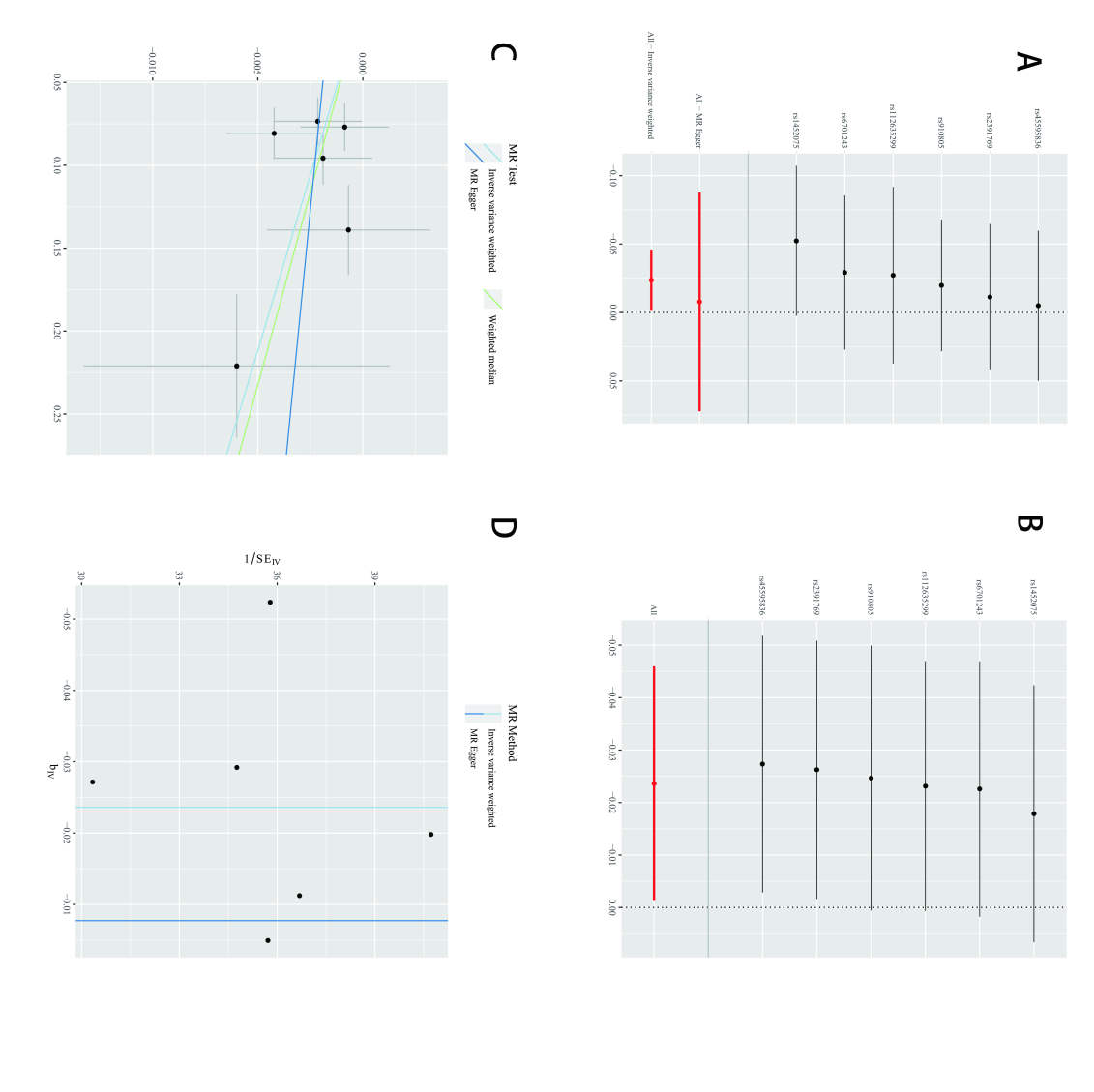


**Supplementary Figure 1. A.** MR estimates of the association between ASD and TL. We found a significant genetic association between ASD and TL (OR = 0.98, 95% CI: 0.96-0.99, P = 0.03). **B.** Leave-one-out plots of ASD and TL. The leave-one-out plot visualizes how the causal estimates (point with horizontal line) for the effect of ASD on TL are influenced by the exclusion of individual SNPs. We did not find that a particular SNP had a significant effect on the results. **C.** Scatter plot of the association between ASD and TL. Three lines reveal the estimated effect sizes by MR methods (inverse‐variance weighted, MR-Egger and weighted median). **D.** Funnel plot on ASD and TL. The funnel plots are symmetric, which shows that the absence of polymorphism.

*
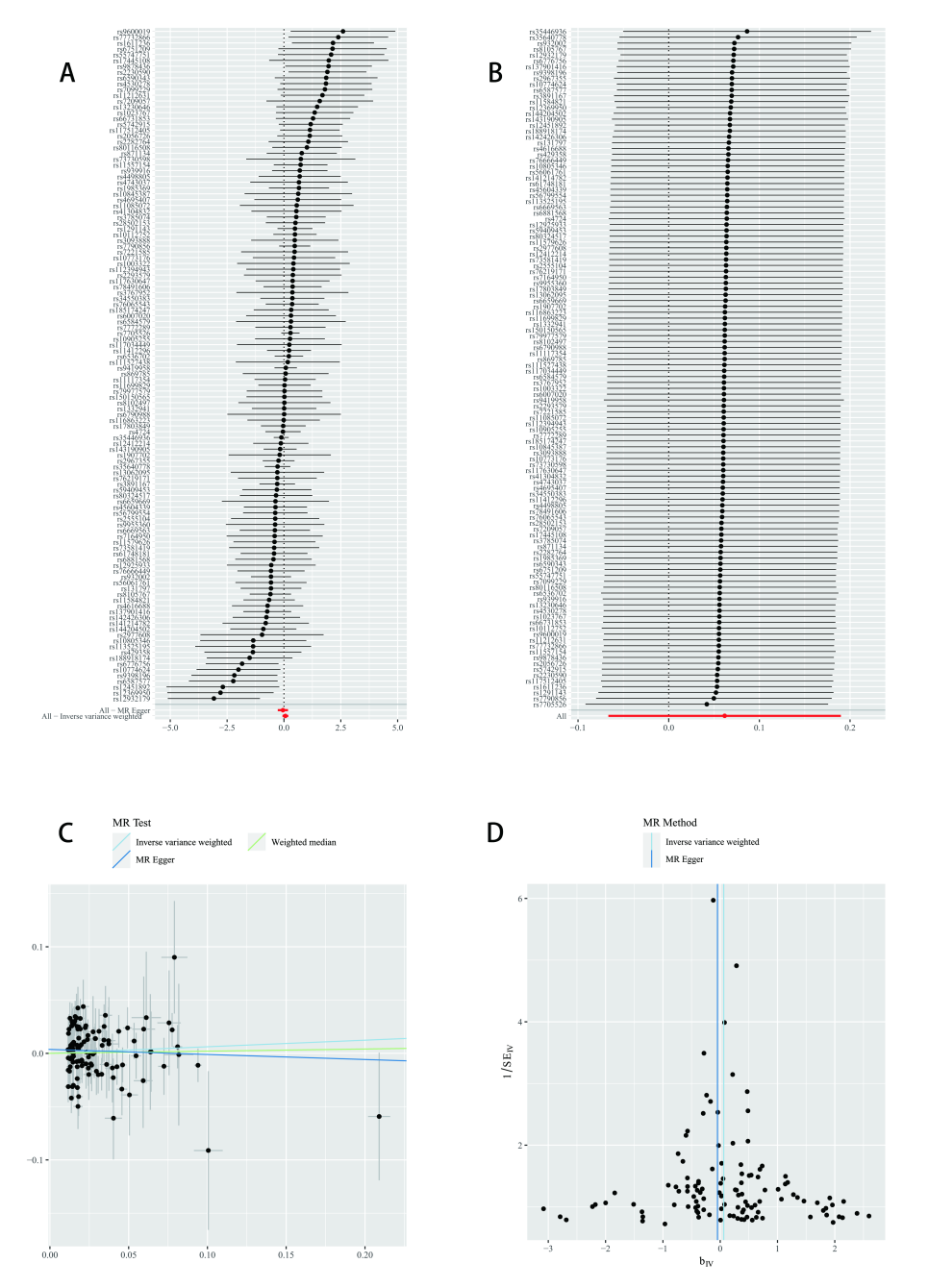
*

**Supplementary Figure 2. A.** MR estimates of the association between TL and ASD. We did not find a significant genetic association between ASD and TL (OR = 1.06, 95% CI: 0.94-1.23, P = 0.35). B. Leave-one-out plots of TL on ASD. We did not find that a particular SNP had a significant effect on the results. **C.** Scatter plot of the association between ASD and TL. **D.** Funnel plot of TL on ASD. The funnel plots are symmetric, which shows that the absence of polymorphism.


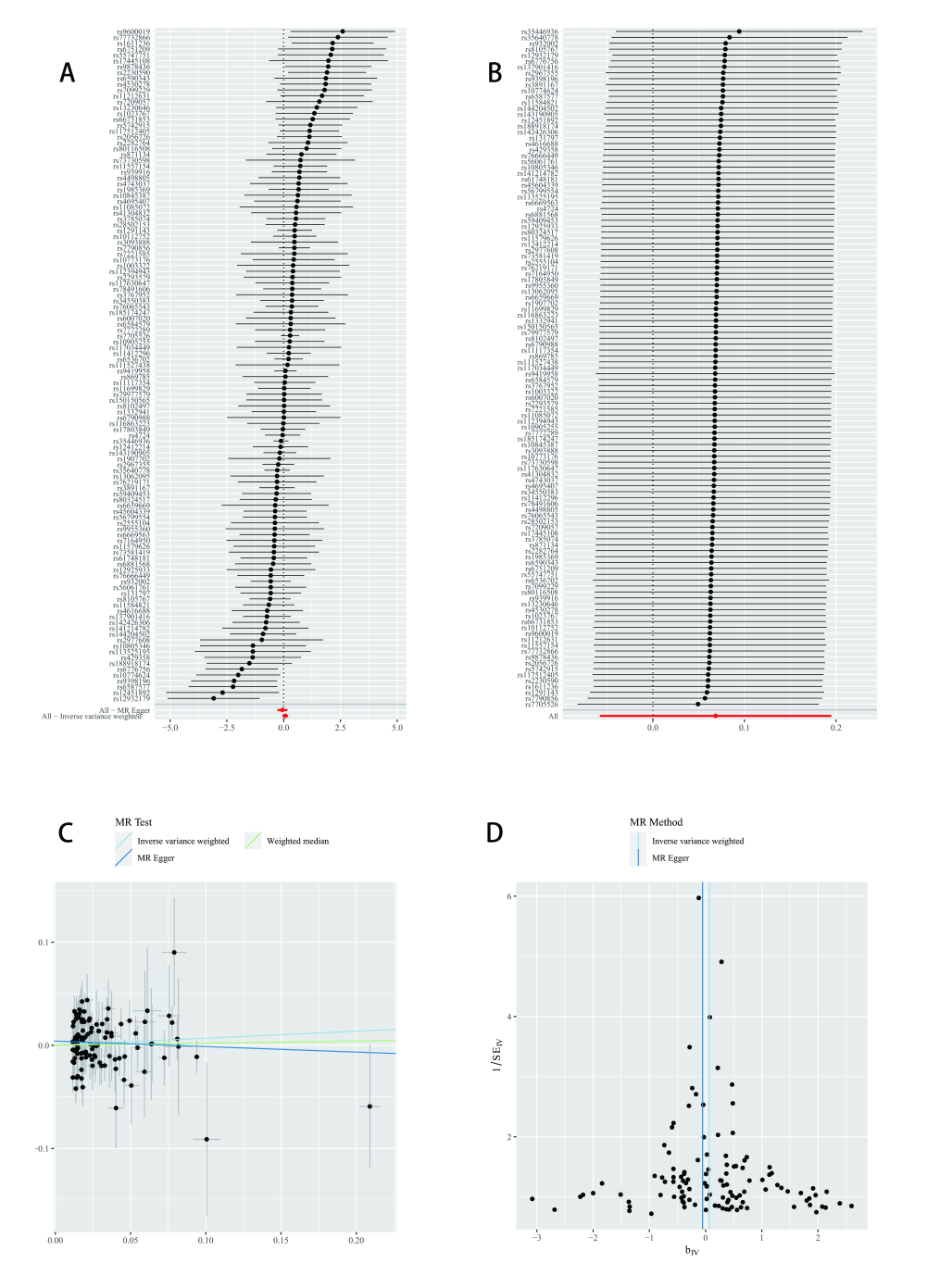


**Supplementary Figure 3. A.** MR estimates of the association between TL and ASD after MR-PRESSO adjustment. We still did not find a significant genetic association between ASD and TL (adjusted, OR = 1.07, 95% CI: 0.94-1.22, P = 0.29). B. Leave-one-out plots of TL on ASD (adjusted). We did not find that a particular SNP had a significant effect on the results. **C.** Scatter plot of the association between ASD and TL (adjusted). **D.** Funnel plot of TL on ASD. The funnel plots are symmetric, which shows that the absence of polymorphism (adjusted).
