# Supplementary figures and images for: Causality between Autism Spectrum Disorder and Telomere Length
Source: Brain Behav. 2025 Feb 19;15(2):e70362. doi: 10.1002/brb3.70362 (PMC11839737; doi:10.1002/brb3.70362)

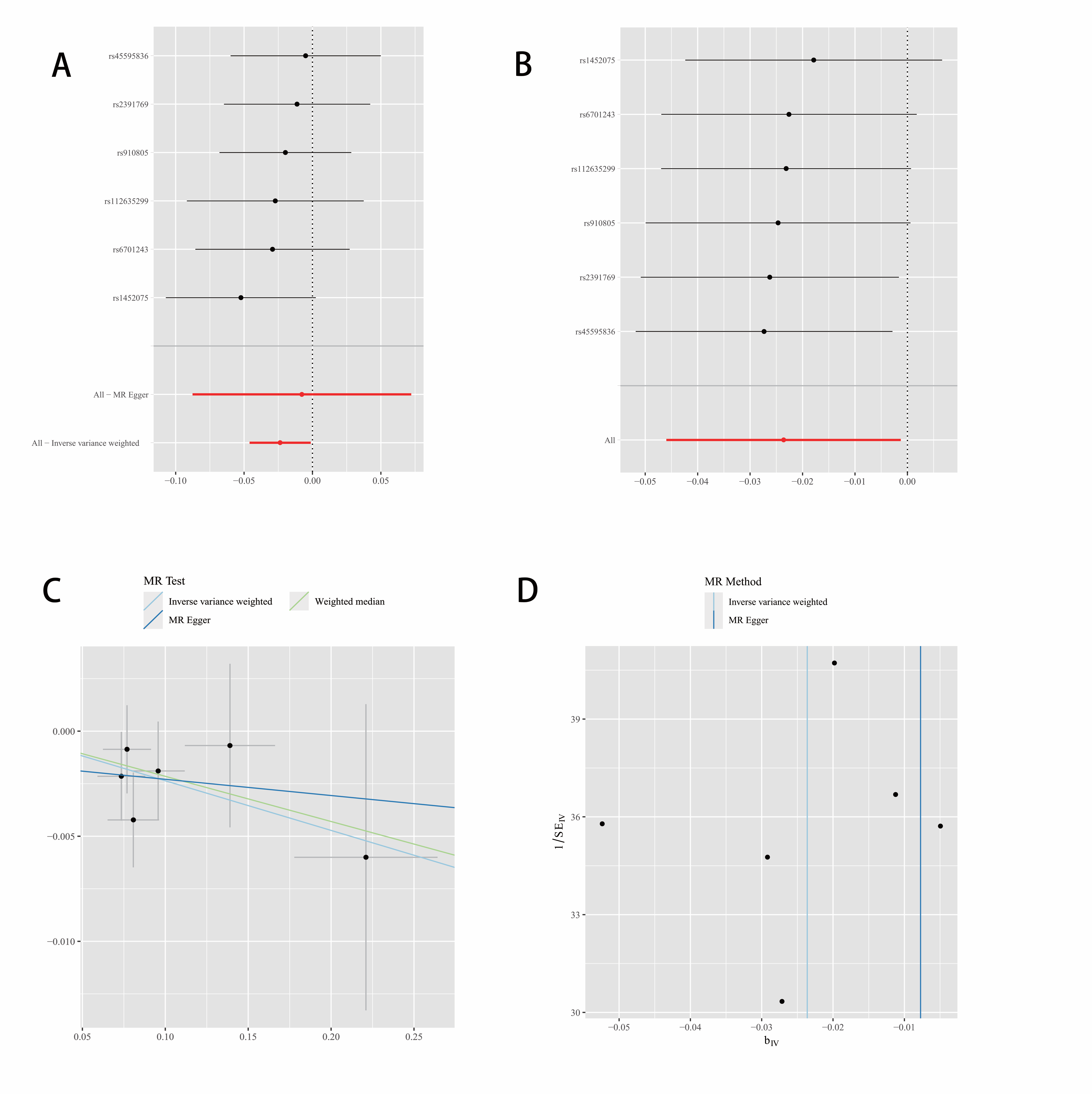

Supplement: Supplementary file 3 — Supporting Information [file BRB3-15-e70362-s003.tif]

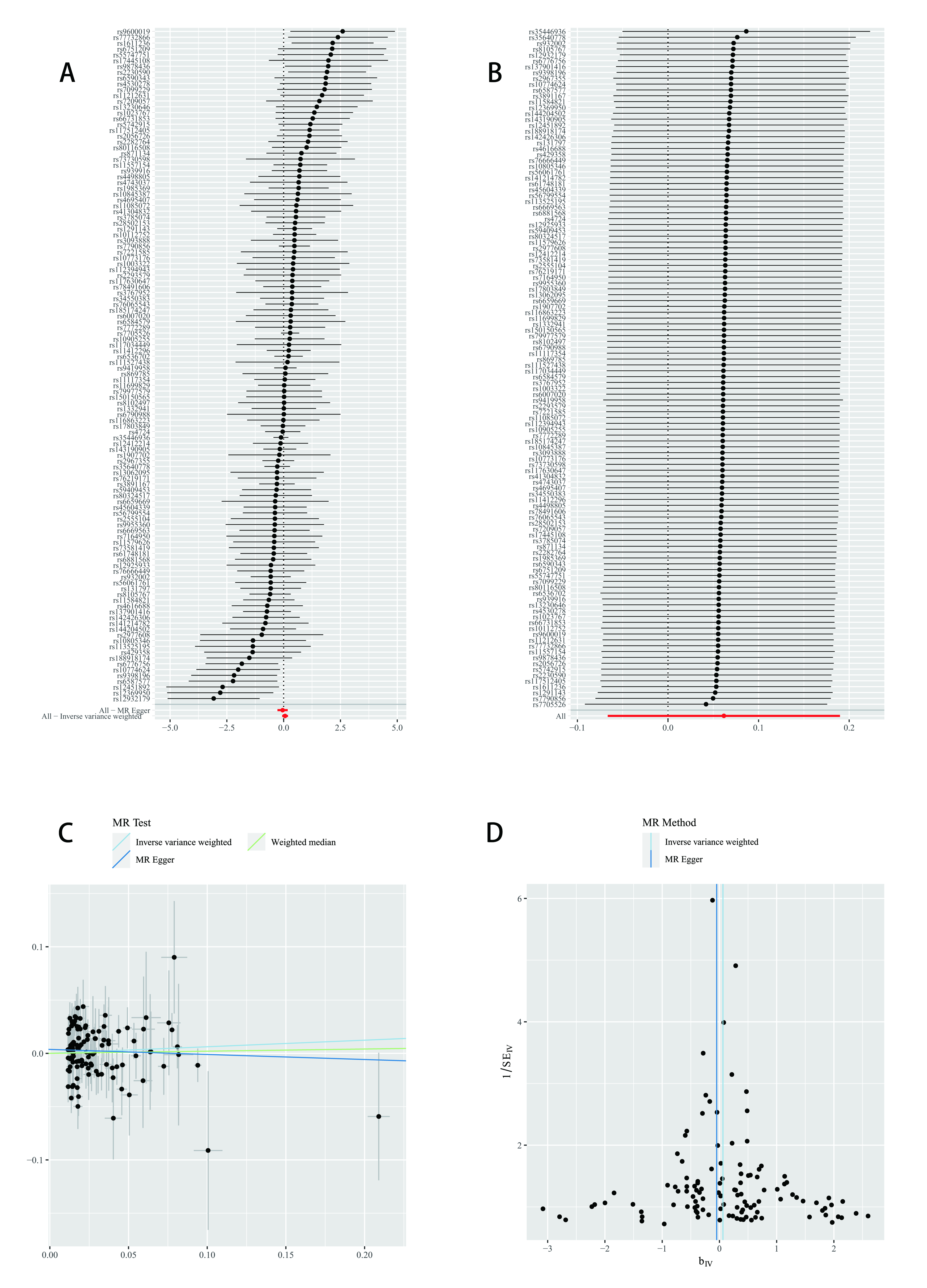

Supplement: Supplementary file 4 — Supporting Information [file BRB3-15-e70362-s005.tif]

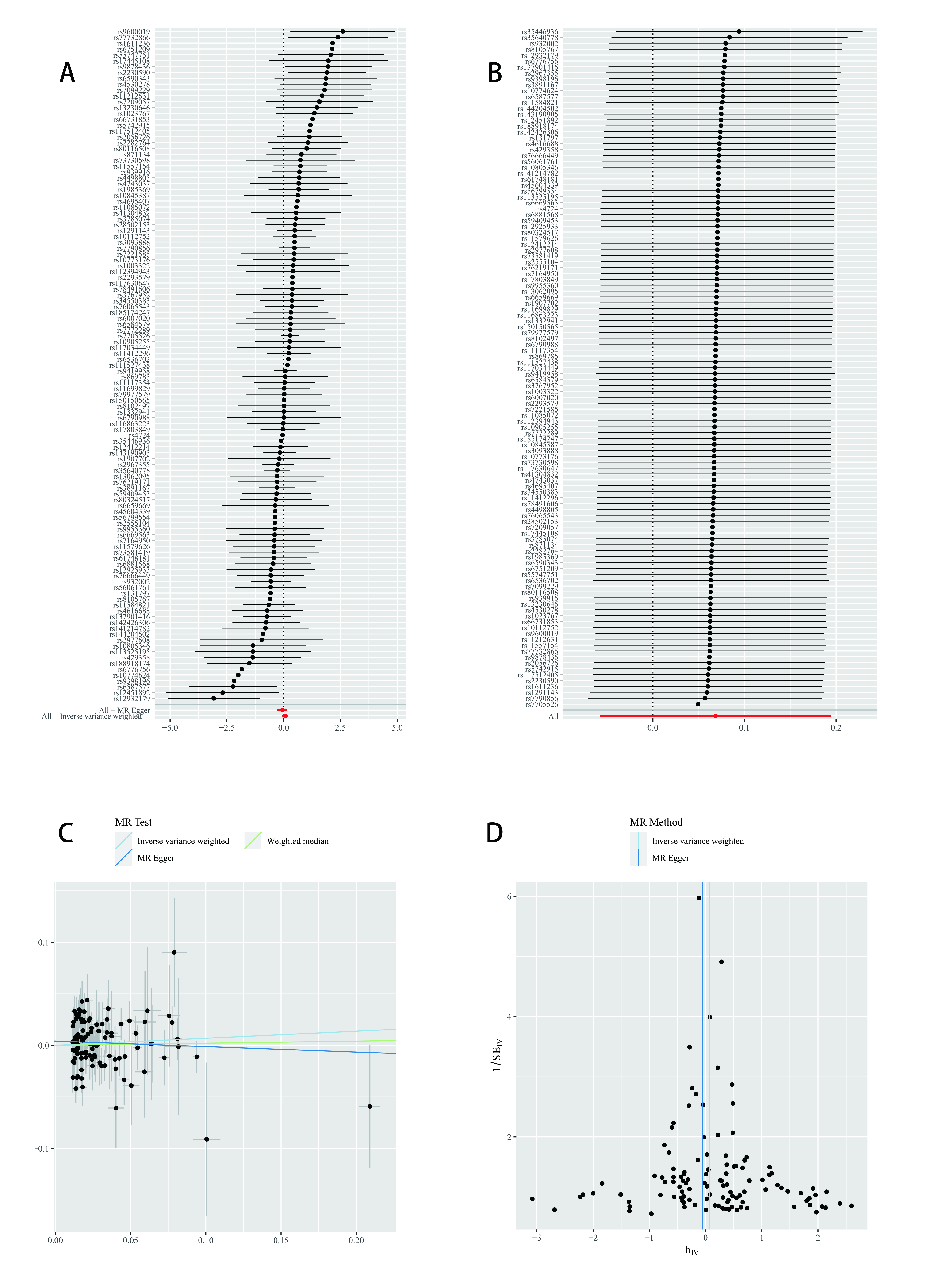

Supplement: Supplementary file 5 — Supporting Information [file BRB3-15-e70362-s002.tif]
